# Application of electrical resistivity tomography (ERT) for rock mass quality evaluation

**DOI:** 10.1038/s41598-021-03217-8

**Published:** 2021-12-08

**Authors:** Muhammad Hasan, Yanjun Shang, He Meng, Peng Shao, Xuetao Yi

**Affiliations:** 1 Key Laboratory of Shale Gas and Geoengineering, Institute of Geology and Geophysics, Chinese Academy of Sciences, No. 19, Beitucheng Western Road, Chaoyang District, Beijing, 100029 People’s Republic of China; 2 College of Earth and Planetary Sciences, University of Chinese Academy of Sciences, Beijing, 100049 People’s Republic of China; 3 Innovation Academy for Earth Science, Chinese Academy of Sciences, Beijing, 100029 People’s Republic of China

**Keywords:** Environmental sciences, Solid Earth sciences, Astronomy and planetary science, Engineering

## Abstract

Rock mass quality evaluation is a challenging task in geotechnical investigations given the natural heterogeneity and the limited data. These investigations mainly depend on the traditional drilling tests. However, such tests are expensive and time consuming, provide point measurements, and cannot be conducted in steep topographic areas, and thus cause uncertainties in the geological model. Conversely, geophysical methods such as electrical resistivity tomography (ERT) are non-invasive, user-friendly, and fast. In this work, we establish empirical correlation between ERT and limited drilling data to obtain rock mass integrity coefficient (Kv). The estimated Kv provides 2D/3D imaging of the rock mass quality evaluation via weathered/unweathered rock and faults detection in order to cover the entire area even where no drilling test exists. Compared with the past geotechnical investigations, our work reduce the ambiguities caused by the inadequate well tests and provide more accurate geological model for infrastructures design. Our work proposes that, in case of sparse borehole data, the established empirical equations can be used to determine Kv along different geophysical profiles via 2D/3D insight of the subsurface. Our approach is applicable in any hard rock setting, and the established correlations can be used in areas even where no well test exists.

## Introduction

Rock mass quality evaluation is a challenging task all over the world. In hard rock terrains, successful construction of the engineered structures mainly relies on bearing capacity of the foundation rocks^1,2^. The bearing strength of these rocks depends on various factors, such as type of rock, weathering degree, mineralogical composition, rock association, faults/fractures, rock deformation and water infiltration etc^3,4^. The failure of engineering infrastructures is mostly caused by the weak bearing capacity of foundation rocks^5^. Thus, a thorough investigation of the subsurface rocks is essential to obtain an accurate geological model for the success of foundation design. The geotechnical sites are investigated for the evaluation of engineering rock quality and fractures/faults^6–9^.

Rock mass integrity coefficient (Kv), rock quality designation (RQD), volume joint number of rock mass (JV) and average joint spacing (dP) are the main geotechnical indices to determine general stability of the subsurface rock mass^10–15^. These parameters are widely used to classify the bearing strength of the engineering rock mass^16^. The rock mass quality parameters, however, are conventionally determined by the rock core samples obtained from the boreholes^1,5,7,14^. The borehole tests provide limited coverage of the vertical measurements along some specific points only, cannot evaluate the subsurface laterally, and are hardly conducted in the steep topographic areas^2,17^. Besides, such approaches are costly and time consuming, and require more equipment^18^. In most cases, the drilling tests of large sites fail to meet the needs of engineers^19^. Hence, the rock mechanical parameters are hard to obtain from the frequent boreholes. Using the traditional geotechnical approaches, it is a difficult task for the planners to obtain an accurate geological model for the engineering structure design. Therefore, a relievable approach is necessary which can determine the geotechnical parameters, reduce an extensive number of drilling tests, and bring a sigh of relief to the planners.

Many researchers have used geophysical methods in their geotechnical investigations^20–33^. Unlike borehole approaches, geophysical methods evaluate the subsurface without any physical disturbance and provide volumetric measurements^34^. Furthermore, such methods are economical, non invasive, rapid and user friendly^35^. Seismic methods are the commonly used geophysical methods in the past geotechnical studies^4,5,36–41^. The seismic detection is likely to be affected by the noise, and cannot delineate the low velocity layer under the high velocity layer and the shallow layer (less than 50 m)^4^. Besides, mostly, the seismic detection consists of 1D/2D probing techniques and the 3D seismic method is still in the early research level^4^. Thus, such methods cannot evaluate the subsurface accurately and leave uncertainties in the interpretation of geological models. However, compared with other geophysical methods, electrical resistivity tomography (ERT) has wide range of resistivity values, shows high correlation between electrical resistivity and lithology of the subsurface layers, provide the required depth of investigation, and evaluates the subsurface via 2D and 3D imaging^42^. Therefore, at present, ERT is one of the leading geophysical methods in geotechnical research.

The correlation of geophysical parameters with aquifer or geotechnical parameters is well known. In the past, many authors established a relationship between geophysical and rock mass quality parameters, since both geophysical and geotechnical parameters are controlled by the same structural heterogeneity including type of lithology/rock, porosity and permeability of rock, weathering degree, water infiltration/saturation (amount of water), fractures/faults, rock association, rock deformation, water–rock interaction and alteration, temperature, and pressure etc^9,11,12,14,16,18,19,36,39,40,43–45^. The correlation between geophysical and aquifer parameters has been widely used in groundwater studies to obtain hydraulic parameters (hydraulic conductivity and transmissivity). However, in geotechnical investigations, such correlation (between geophysical and rock mechanical parameters) has not yet been used to determine the rock mass quality parameters (Kv and RQD). In such studies, the relationship between geophysical and rock mechanical parameters was used to evaluate the subsurface layers (i.e., weathered and unweathered layers) only using specific values range of geophysical parameters (electrical resistivity and seismic velocity). Therefore, low coverage of Kv and RQD obtained from the limited boreholes cause uncertainties in the interpretation of subsurface geological model.

In this work, we have used the empirical proportions between geophysical and geotechnical parameters to obtain Kv and RQD for large coverage of area. In our approach, we propose that only the certain essential locations of the study area could be selected for drilling tests, while geophysical survey such as ERT would be performed to cover the entire project site. Via empirical correlations, geotechnical parameters (Kv and RQD) measured from the limited boreholes would be integrated with the inverted resistivity obtained from the selected ERT data points near the drilling tests. Then, the rock mass quality parameters would be estimated by the obtained empirical equations using the inverted resistivity of all ERT data points. By this way, the rock mechanical parameters are obtained along the same ERT profiles. Thus, the subsurface is thoroughly evaluated via 2D and 3D imaging of Kv or RQD for different types of rocks such as the weathered/crushed rock and the integral or unweathered/fresh rock over the entire investigated area even where no drilling data exists.

**Figure 1:**
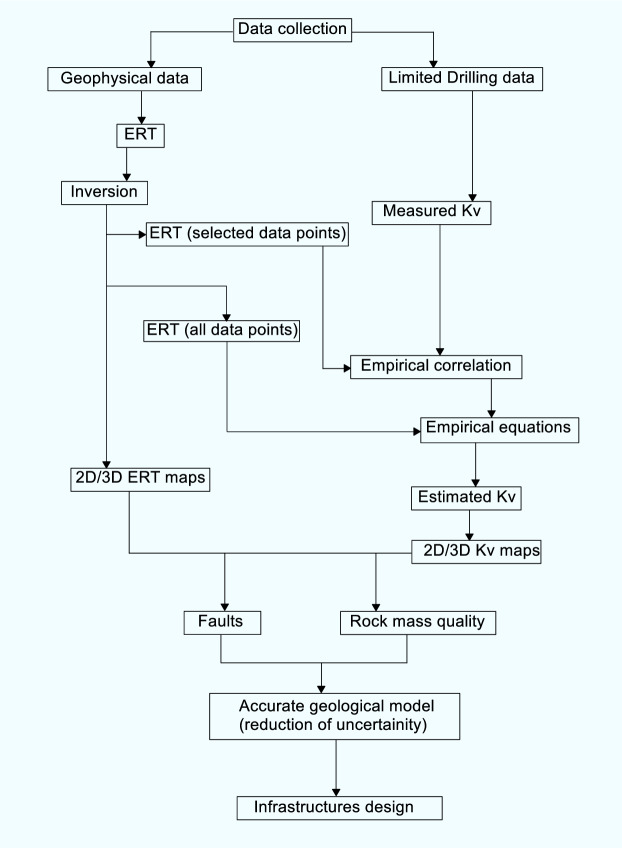
A flowchart to summarize the efforts to obtain an accurate geological model using geophysical approach for infrastructures design.

This work is a rare contribution of the application of non invasive and economical ERT towards the geotechnical investigation, which not only reduces number of drilling tests but also provides 2D and 3D imaging of geotechnical parameters in order to accurately obtain geological models for development of engineering structures. Moreover, Kv has not been estimated via empirical correlation between drilling tests and ERT. Our primary goals were: (1) to reduce the ambiguities in the subsurface geological models caused by natural heterogeneity and the limited data; (2) to bridge the gaps between the inadequate well data and the accurate geological model; (3) to assess the subsurface geological models for rock mass quality evaluation and faults detection via a thorough 2D/3D imaging of ERT and Kv models; (4) to introduce ERT (coupled with available boreholes) as the best alternative approach of the traditional techniques to obtain the rock mechanical parameters by reducing significant number of expensive drilling tests; and (5) to provide scientific basis for infrastructures design in the hard rock sites. Important steps of this work are shown by a flowchart in Fig. 1.

**Figure 2:**
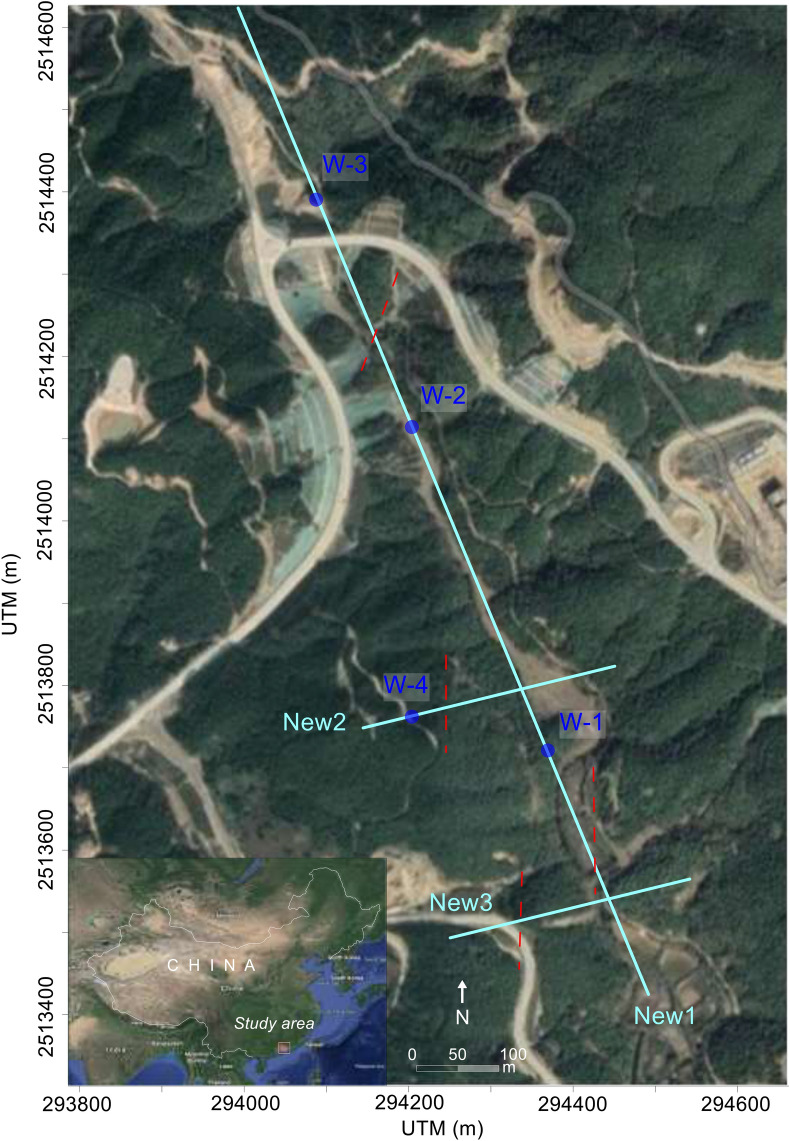
Location of the investigated area, including 3 geophysical profiles New1, New2 and New3 (light blue lines), boreholes W1-4 (blue dots), and the traced faults (red dashed lines in different directions). The map was created on the basis of Google Earth (https://earth.google.com/) by M. Hasan in the CorelDRAW 12.0 Graphic program.

This investigation was carried out in South of Huizhou, Guangdong province, China for the successful development of infrastructures (Fig. 2). Based on the stratigraphic setting, the project site is located in the South China Fold System and the South China Stratigraphic Area. Geomorphologically, the study area is divided into several units such as the East High Mountains, the Middle Green Hills and the Southwest Hills at the coast of South China Sea. Tuff rocks are the most dominant rocks in the investigated area, which belong to the magmatic rocks of Lower Jurassic and acidic volcanic rocks of Upper Jurassic^46^. Based on highly heterogeneous setting of the study area, a detailed study of the subsurface is necessary prior to the construction of engineered structures.

## Methods

### ERT survey

In ERT surveys, electrical resistivity is measured (in Ωm), which is the inverse of electrical conductivity. The basic principle of ERT is based on the varying electrical conductivity of the subsurface materials, which depends on many factors, such as rock type, porosity, permeability, connectivity of pores, temperature, salinity, cation exchange capacity, clay content, nature of the fluid/water, weathering degree, fractures/faults, rock association, rock deformation, water–rock interaction and alteration, etc^3,9,42^. Generally, a wide range of electrical resistivity for most materials suggests the varying water content. In hard rock terrains, electrical resistivity can vary over a large range depending on weathering degree, water saturation, etc^18^. Electrical resistivity decreases with increasing the weathering degree and water content. Hence, a contrast in resistivity values of the saturated weathered/fractured rock and fresh/unweathered rock is clearly observed^9^. 2D/3D ERT is a modern form of the conventional 1D VES (vertical electrical soundings) method, which provides high resolution imaging of the subsurface both laterally and vertically^42^. The modern electrical surveys are conducted using the most efficient and non destructive instruments, which can provide measurements of ERT, IP (induced polarization) and SP (self-potential). IP measures chargeability which is useful when there is little contrast in resistivity of the subsurface lithologies, such as resistivity shows low value for both water and clay content but IP suggests high value for clay than water. IP is the most commonly used electrical method in mineral exploration for sulfides detection. SP is useful to map groundwater flow path. However, compared with other electrical methods (IP and SP), ERT has an advantage of having a wide range of resistivity values and is the most widely used geophysical method to map more accurate geometry of weathered/fractured and unweathered/fresh rock. Therefore, ERT has been widely used as the most suitable electrical method to make the correlation of electrical resistivity with other parameters (hydraulic and geotechnical parameters)^9,18^. ERT surveys are carried out by large number of apparent-resistivity measurements^47,48^. The apparent resistivity data are acquired through the injection of electric current into the ground using two current electrodes, and another pair of potential electrodes is used to measure the resulting potential difference along a 2D profile. The investigation depth can be improved (increased) by increasing the electrodes spacing; however, this will reduce the resolution of subsurface model^49^. ERT provides an image with the distribution of subsurface resistivity. Based on resistivities of different subsurface materials/rocks, the resistivity image can be converted into the subsurface image having different lithologies/rocks^18^. The interpretation of resistivity results is called as pseudosection^50^. ERT data acquisition is performed via various electrode arrays (e.g., Wenner, Schlumberger, dipole–dipole, pole–dipole and pole–pole). Different electrode arrays provide different investigation depths and subsurface resolution. The electrode array is selected based on many factors such as signal strength, resistivity sensitivity to lateral/vertical variations in the subsurface, depth of investigation, and the lateral coverage of resistivity data^51^.

ERT survey was conducted by a Terrameter SAS 4000 (ABEM, Inc.). The apparent resistivity measurements were acquired using various electrodes (non-polarizing) connected to a multi-core cable. The multi-electrode imaging system (48 electrodes) uses a multi-function electrical instrument and a multi-electrode convertor. The same instrument (Terrameter SAS 4000) can be used to obtain measurements of three electrical methods (ERT, IP and SP) using different modes. In this investigation, the imaging system was turned into resistivity mode only to perform the apparent resistivity measurements. We used the pole–dipole array for ERT data acquisition since it provides higher signal strength, more sensitivity to the vertical structures and large investigation depth^52^. Furthermore, it is less influenced by the remote-pole position, and is more suitable for the delineation of weathered/fractured rock^9^. Asymmetrical effects of the pole–dipole were removed by integrating the forward and reverse measurements. The data quality was improved by enhancing signal strength using the stacking procedure^53^. Roll-Along mode with different layouts was used to increases the profile length. ERT survey was performed along three profiles namely New1, New2 and New3 using a fixed electrode spacing of 5 m, a total of 387 electrodes, and a total profile length of 1920 m. We performed profile New1 with a profile length of 1300 m for 261 electrodes, profile New2 using 65 electrodes and profile spread of 320 m long, and profile New3 for 61 electrodes and 300 m profile length.

The apparent resistivity measured in the field assumes a homogeneous subsurface. Therefore, in order to obtain true resistivity of the subsurface materials, the apparent resistivity must be inverted using the inversion software^18^. Therefore, the data sets of apparent resistivities obtained along each profile were processed via the algorithms and programming code of RES2DINV^54^. This software uses the nonlinear optimization program to generate a 2D ERT pseudosection. The inversion software uses the smoothness-constrained least squares with L2-norm^55^. The data quality was enhanced via robust constraint. RES2DINV tries to minimize the RMS (root mean square error) by adjusting resistivities of the model blocks. RMS defines the difference between the measured and calculated apparent resistivities. Each 2D ERT model was generated for RMS below 5% after 8 iterations. The inversion program was performed using the standard technique of Gauss Newton optimization. The data acquisition of ERT method is a 2D configuration process. The 2D ERT inversion was carried out along each profile using RES2DINV. Then, the 2D ERT data of three profiles were converted into 3D ERT inversion format using RES3DINV. Afterwards, for better comparison of geophysical and geotechnical parameters via similar mapping of ERT and Kv, ERT inversion data and the obtained Kv data were used by Geosoft or SKUA-GOCAD software for further reconstruction of ERT/Kv models^56–59^.

### Rock mass integrity coefficient (Kv)

Rock mass integrity coefficient (Kv) also known as the fracture coefficient of rock mass is one of the most efficient geotechnical indices used in rock mass quality evaluation^15^. Conventionally, Kv is measured from the rock core samples of the boreholes. It is calculated by the square of the ratio of the acoustic P-wave velocity of the same rock mass to the P-wave velocity of the rock block^15,60^. The following equation is used to measure Kv from the drilling data:

1 $$Kv = \left( {\frac{Vpm}{{Vpr}}} \right)^{2}\quad ( {0 \le Kv \le 1} )$$

where Kv indicates the rock mass integrity index ranging between 0 and 1, Vpr is velocity of P-wave (in km/s) for the intact rock, and Vpm shows the acoustic velocity of P-wave (in km) for the rock mass. The acoustic velocity of P-wave (Vpm) is obtained by in-situ measurements, while the velocity of P-wave (Vpr) is acquired from the rock core samples of drilling tests. The rock mass acoustic P-wave velocity (Vpm) mainly depends on composition of rock, structural characteristics, discontinuities of rock mass, cementation of joints and groundwater occurrence. Rock mass integrity index (K_V_), compared with other integrity coefficients such as rock quality designation (RQD), average joint spacing (dP) and volume joint number of rock mass (JV), is the most reliable geotechnical coefficient in rock engineering for rock mass quality evaluation^15^.

**Figure 3:**
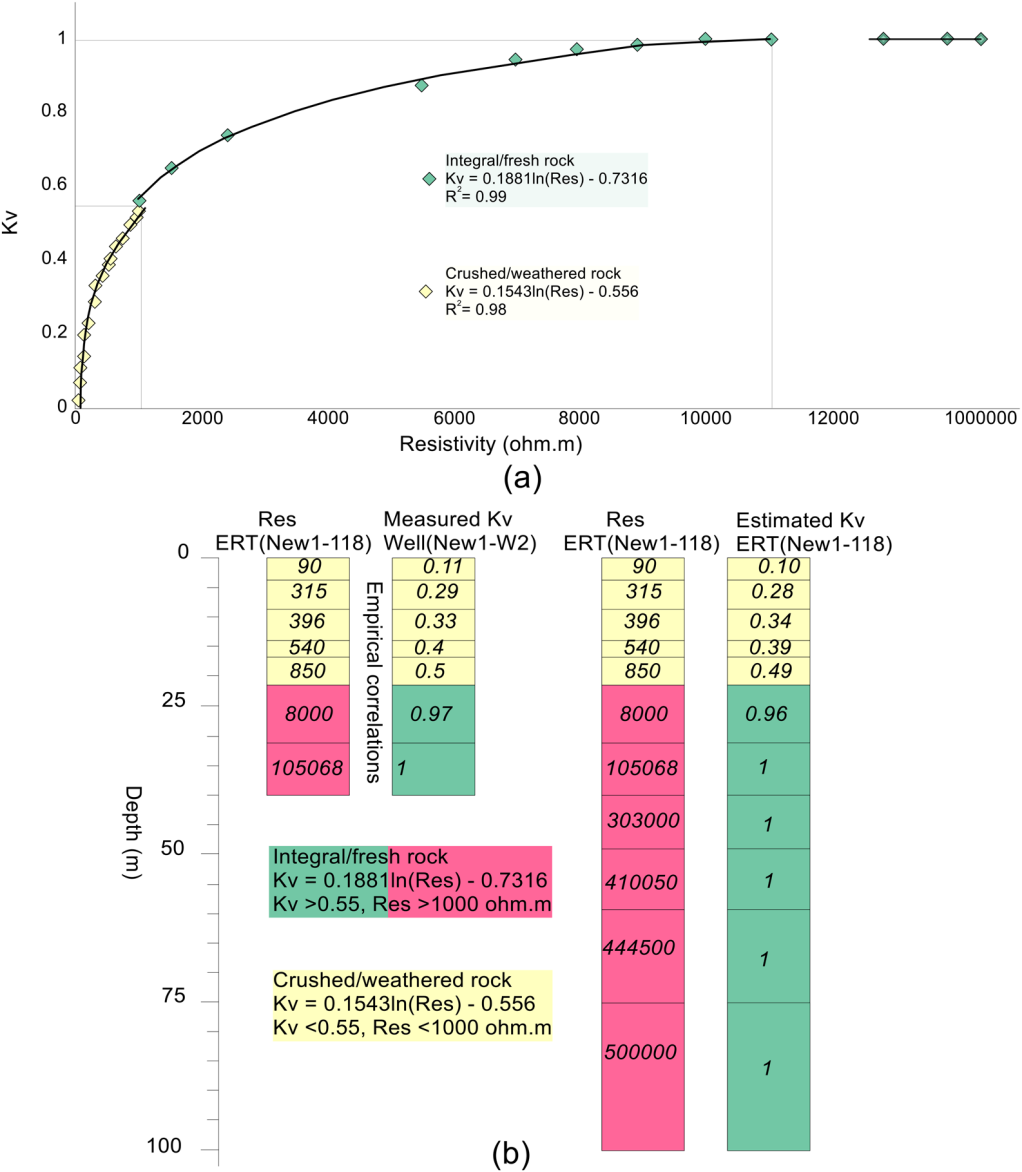
(**a**) Empirical correlations between the inverted electrical resistivity acquired from the selected ERT data points and the rock mass integrity coefficient (Kv) measured from the limited drilling test data. (**b**) An example of the correlation process between the selected ERT point NEW1-118 and Kv of borehole well 2.

Many authors suggest that useful correlations can be established between geophysical and geotechnical parameters^12,14,16,19,36,39,43^. Firstly, Eq. (1) was used to measure Kv for the data obtained from 4 boreholes. From four well points, we acquire a total of 28 kV values at different depths. Then, we performed empirical correlation between the inverted resistivity values (obtained from the selected ERT data points near the well points) and the measured Kv (Fig. 3a) to obtain Kv for all ERT data points along three profiles over the entire study area even where no borehole existed. A total of seven Kv values at different depths (between 5–40 m) were acquired from each borehole. Hence, we get a total of 28 kV values from 4 wells for maximum depth of 40 m. We make the correlation of 4 drilling wells with four ERT data points (out of total 387) along three profiles, such as New1-41 (41st ERT data point along profile New1) near borehole 1, New1-118 (118th ERT data point along profile New1) near borehole 2 (Fig. 3b), New1-212 (212nd ERT data point along profile New1) near borehole 3, and New2-15 (15th ERT data point along profile New2) near borehole 4. By this way, the empirical correlations were established between 28 values of the inverted resistivity (obtained from 4 ERT data points at different depths corresponding to the nearby drilling wells) and 28 kV values (measured from 4 wells at the same depths) (Fig. 3a). First of all, we obtained only a single empirical equation for both weathered and fresh rock. However, we observed that such equation provides error for the estimation of Kv especially for low and high resistivities, hence it causes ambiguities in rock mass quality evaluation. Such uncertainties in the subsurface geological model were reduced via empirical equation for each type of rock (Fig. 3a).

2 $$Kv = 0.1543{\text{ln}} \, ( {Res.} ) - 0.556$$

Equation (2) was used to evaluate the crushed/weathered rock for resistivity less than 1000 Ωm and Kv between 0–0.55.

3 $$Kv = 0.1881{\text{ln}} \, ( {Res.} ) - 0.7316$$

Resistivity greater than 1000 Ωm was used in Eq. (3) to estimate Kv ranging from 0.55 to 1 for the integral/fresh rock.

The above equations were used for resistivities of all ERT data points to obtain Kv for the rock mass quality evaluation over the entire area. One of the correlation points (e.g., between ERT point New1-118 and borehole 2) is shown in Fig. 3b. Therefore, compared with 28 kV values (measured from 4 borehole points for maximum depth of 40 m), 7740 values of Kv [estimated along three profiles for a total of 387 ERT data points and 100 m depth using Eqs. (2) and (3)] provide far better insights into the subsurface for rock mass quality evaluation. The obtained empirical equations can be used for the estimation of Kv in the areas with similar setting where no drill data is available. Furthermore, the similar methodology can be followed to establish empirical equations for any specific area with any setting.

## Results

### Correlation between geophysical and boreholes data

**Figure 4:**
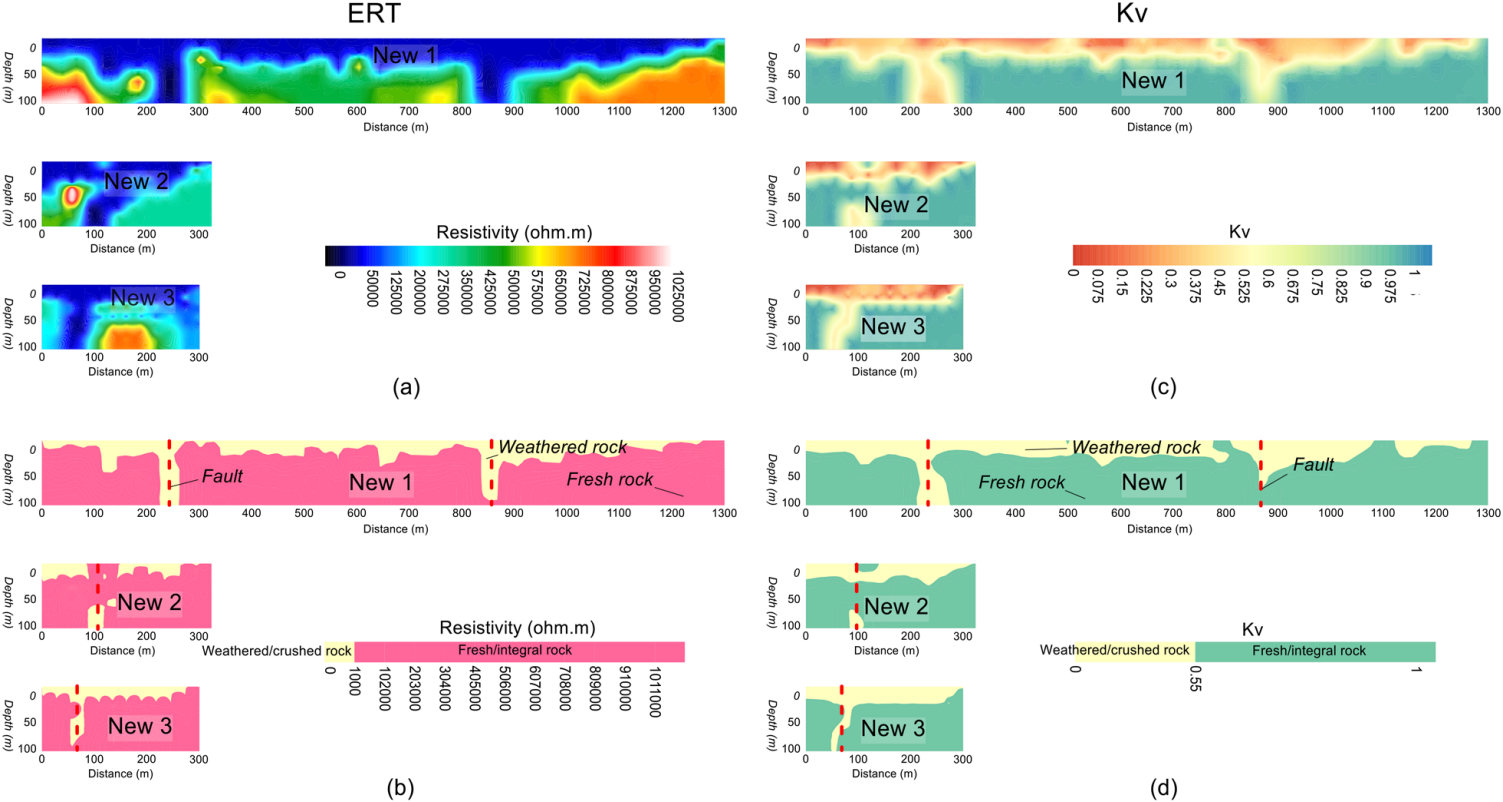
(**a**) 2D ERT models along three profiles New1, New2 and New3 (resistivity increasing from dark blue to red white on a color scale). (**b**) Interpretation of a for the weathered/crushed rock (yellow color) and the fresh/integral rock (red color). (**c**) 2D Kv models along profiles New1, New2 and New3 (Kv increasing from red to blue on a color scale). (**b**) Interpretation of c for the weathered/crushed rock (yellow color) and the fresh/integral rock (green color).

Correlation between the ERT data and the borehole information constrained the subsurface into a two layered model. Such calibration was carried out using the inverted resistivity values acquired from the selected four ERT data points near the boreholes, Kv measured from four drilling wells and the local geological knowledge (Table 1). The subsurface was evaluated by two discrete layers with overall resistivity varying from 0 to 1,025,000 Ωm and Kv from 0 to 1. Based on resistivity less than 1000 Ωm and Kv ranging from 0 to 0.55, the near-surface upper layer was interpreted as the weathered/crushed rock. The bottom layer, underlying the first layer of the weathered rock, was delineated as the integral/fresh rock having resistivity greater than 1000 Ωm and Kv between 0.55–1.00. The calibration results suggest that Kv increases with increasing resistivity from top surface to the bottom layer (e.g., with depth). The near-surface layer of the weathered/crushed rock was interpreted as the rock mass of poor quality, which suggests unsuitable places for the development of infrastructures. The bottom layer of the fresh/integral rock was delineated as the rock mass of good quality, which offers the most appropriate locations for the engineered structures design in the project site. The 2D ERT models along profiles New1, New2 and New3 are shown in Fig. 4a. Based on the correlation between geophysical and well data, the 2D ERT profiles were interpreted for a two-layered model of the weathered and fresh rock (Fig. 4b). The 2D Kv models were obtained along the same ERT profiles via empirical correlations between the ERT and well data (Fig. 4c). The 2D Kv models interpreted for the weathered/crushed and integral/fresh rock are shown in Fig. 4d.

**Table 1:** Correlation between ERT and well data using the specific values range of resistivity and Kv for rock mass quality evaluation.

Resistivity (Ωm)	Kv	Rock mass quality	Site suitability for infrastructures
< 1000	0–0.15	Weathered/crushed rock	Unsuitable
> 1000	0.55–0.75	Fresh/integral rock	Suitable

### Rock mass quality imaging using ERT

**Figure 5:**
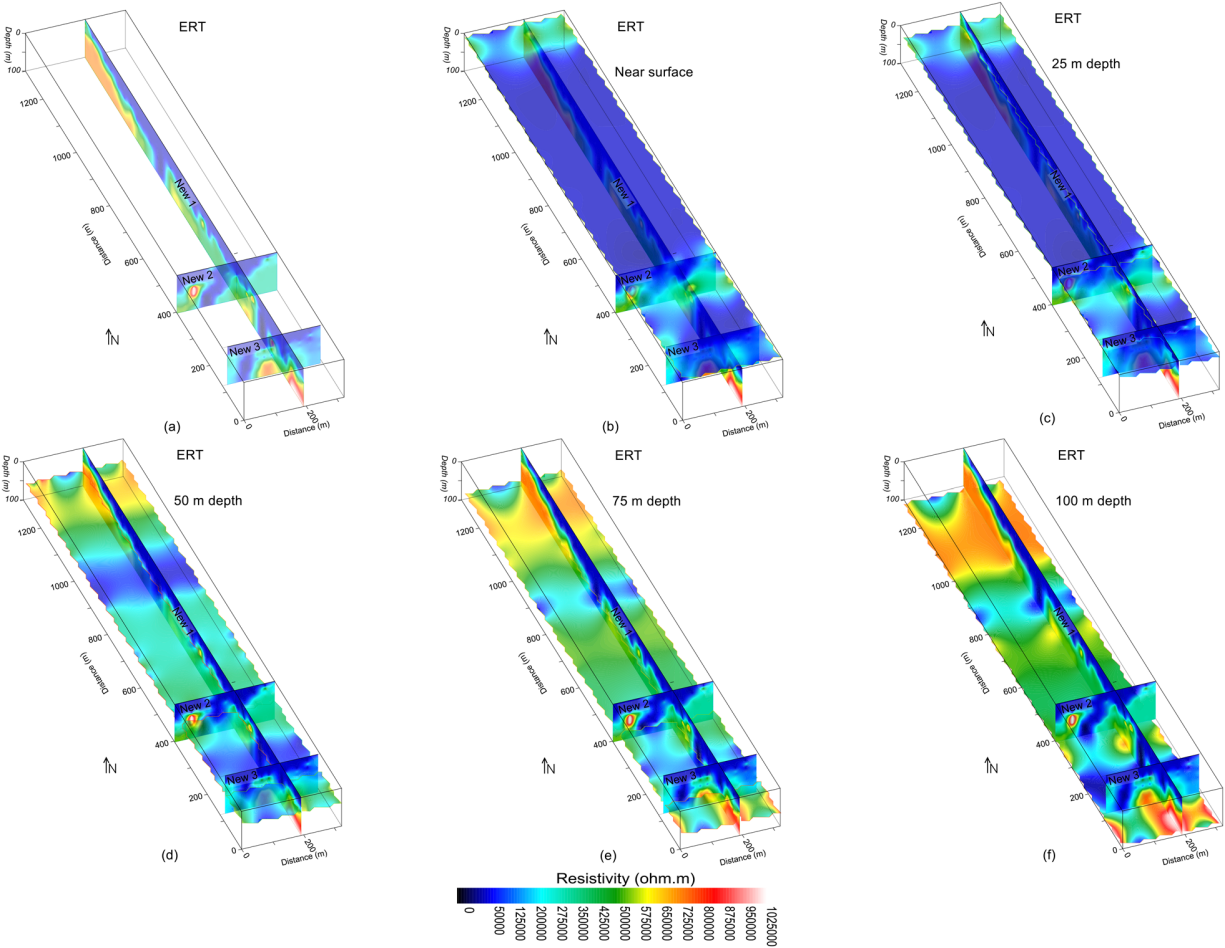
Resistivity imaging (increasing from dark blue to red white on a color scale) for: (**a**) the integration of three ERT profiles; (**b**) ground surface including (**a**); (**c**) 25 m depth including (**a**); (**d**) 50 m depth including (**a**); (**e**) 75 m depth including (**a**); and (**f**) 100 m depth (bottom) including (**a**).

**Figure 6:**
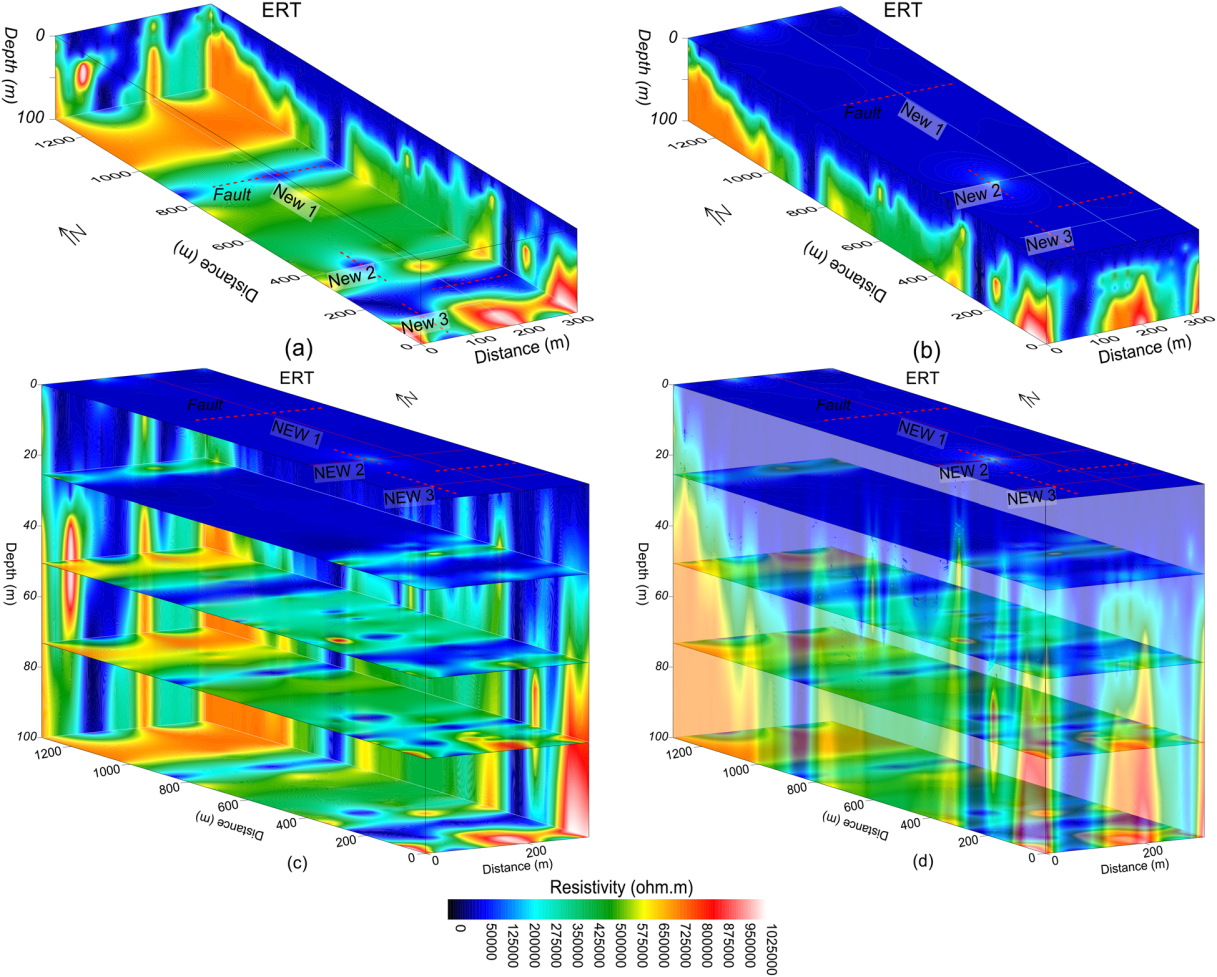
Resistivity imaging (increasing from dark blue to red white on a color scale) along three profiles (red lines) including faults (dashed red lines) for: (**a**) the inner view of 3D ERT, (**b**) the outer view of 3D ERT, (**c**) different depths from ground surface to 100 m with 3D ERT (inner view), and (**d**) different depths from ground surface to 100 m with 3D ERT (outer view).

**Figure 7:**
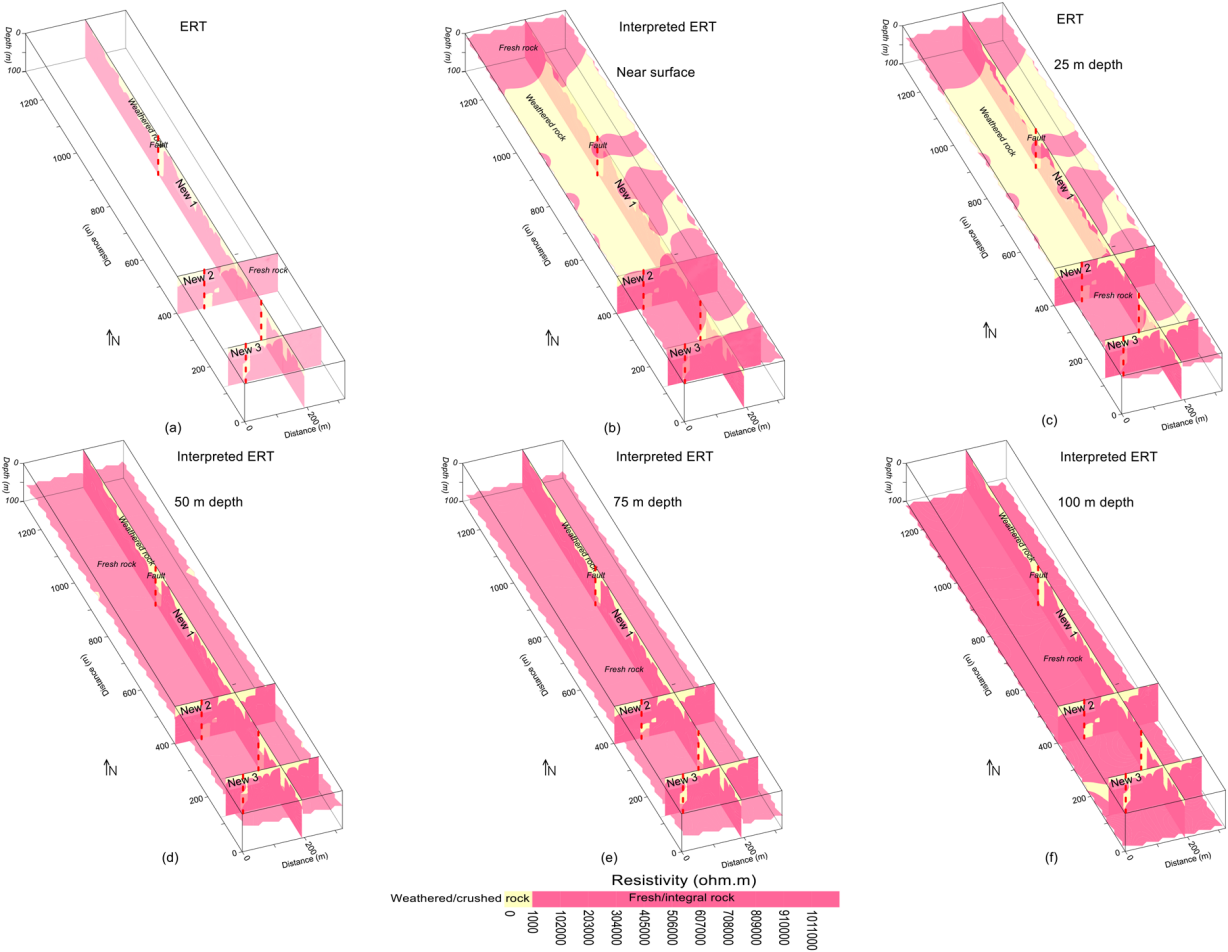
Resistivity imaging interpreted by crushed/weathered rock (yellow color), integral/fresh rock (red color) and faults (dashed red lines) for: (**a**) the integration of three ERT profiles; (**b**) ground surface including (**a**); (**c**) 25 m depth including (**a**); (**d**) 50 m depth including (**a**); (**e**) 75 m depth including (**a**); and (**f**) 100 m depth (bottom) including (**a**).

**Figure 8:**
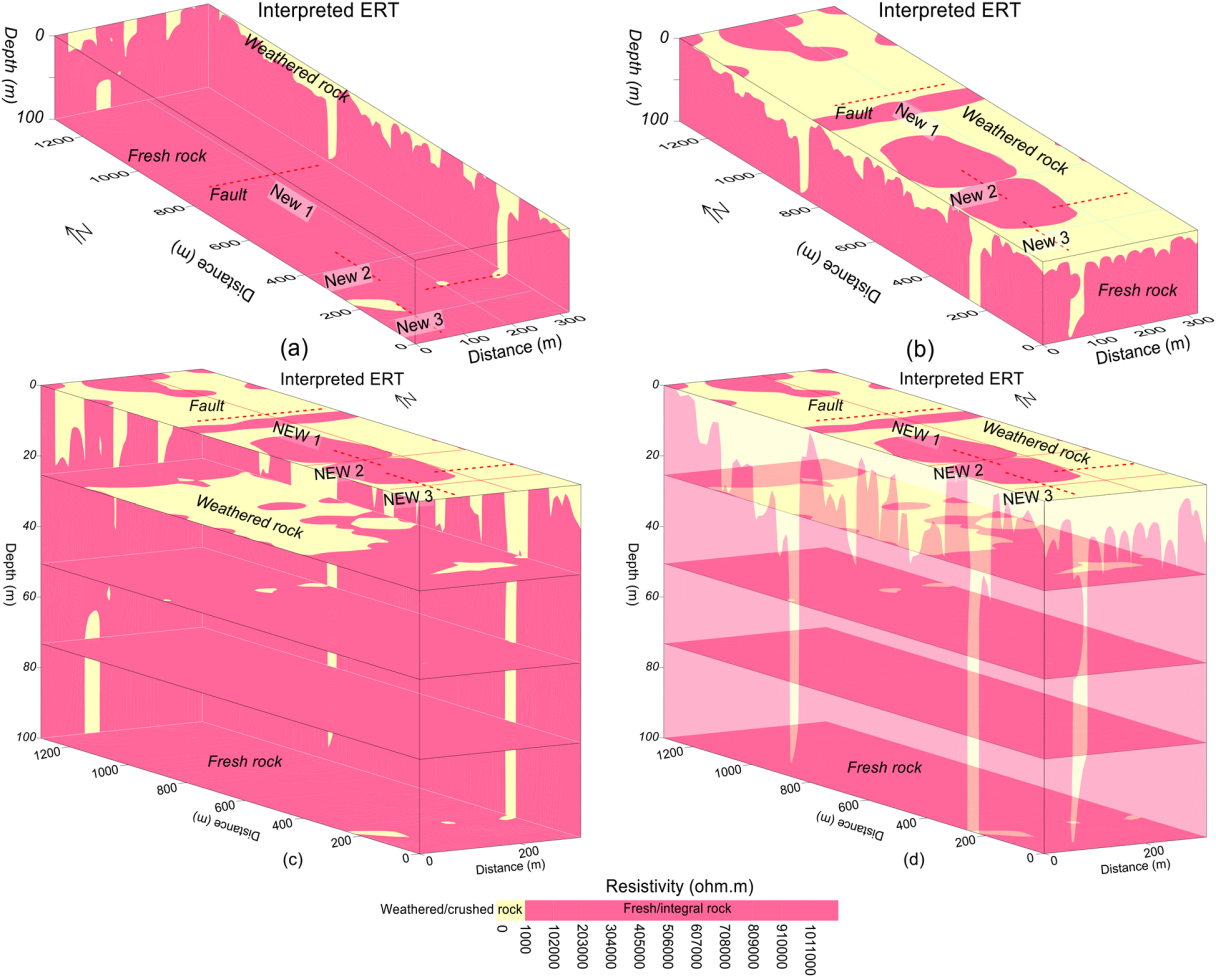
Resistivity imaging along three profiles (red lines) interpreted by the crushed/weathered rock (yellow color) and the integral/fresh rock (red color) and the faults (dashed red lines) for: (**a**) inner view of 3D ERT, (**b**) outer view of 3D ERT, (**c**) different depths including 3D ERT (inner view), (**d**) the outer view of 3D ERT with different depths.

A thorough imaging of the subsurface via the inverted resistivity of ERT along three profiles New1, New2 and New3 is shown in Figs. 5 and 6. The 2D ERT models were interpreted for the weathered/crushed rock and fractures/faults zones using low resistivity, and the integral/fresh rock for high resistivity (Figs. 7 and 8). The resistivity varies from 18 to 1,100,000 Ωm over the entire investigated site. The high resistivity values are found along profile New1, whereas profile New3 shows low resistivity values compared with other profiles. The integrated 2D ERT models (Fig. 5a), including the resistivity imaging at different depths (e.g., 0 m, 25 m, 50 m, 75 m and 100 m) (Fig. 5b–f) provide clearer view of the subsurface, and reveal that mostly the low resistivity zones are located in the central parts, whereas the subsurface zones with high resistivity are dominant in the northwest and southeast of the study area. The 3D ERT mapping (Fig. 6) suggests that the resistivity value increases with depth. The bottom is dominant with the high resistivity (Fig. 6a), whereas mostly the ground surface is revealed with low resistivity (Fig. 6b). Figure 6c,d reveals that most of the low resistive zones are delineated up to 20 m depth, while the high resistive rocks are dominant below 20 m depth.

Based on correlation between ERT and borehole data, the subsurface was interpreted for a two-layered model of the weathered/crushed rock (completely, relatively and poorly weathered/crushed) with resistivity less than 1000 Ωm and integral/fresh rock (relatively and completely integral rock) for resistivity greater than 1000 Ωm (Figs. 7 and 8). The ERT profiles are mostly dominated by the fresh rock of good quality. However, some deep weathered zones are also delineated in the ERT models, i.e., two such zones along profile New1 at 250 m and 850 m distance, one zone along profile New2 at 100 m distance, and another zone at 70 m distance along profile New3 (Figs. 4b and 7). The interface between the weathered and fresh rock is delineated at an average depth of 20 m along all profiles. The interpreted 2D and 3D ERT models suggest that mostly the weathered rock of poor quality is found near the ground and 25 m depth (Figs. 7a–c and 8b–d), whereas the fresh/integral rock is dominant at 50 m, 75 m and 100 m depths (Figs. 7a, d–f and 8a, c, d). Hence, the intensity of weathered/crushed rock decreases with an increase in depth. The weathered/fractured zones (poor rock mass quality) delineated by the interpreted ERT imaging should be avoided in the infrastructure design. However, the places other than the weathered/crushed rock along the ERT profiles are suggested as the most suitable locations for the development of infrastructures.

### Rock mass quality evaluation using Kv

**Figure 9:**
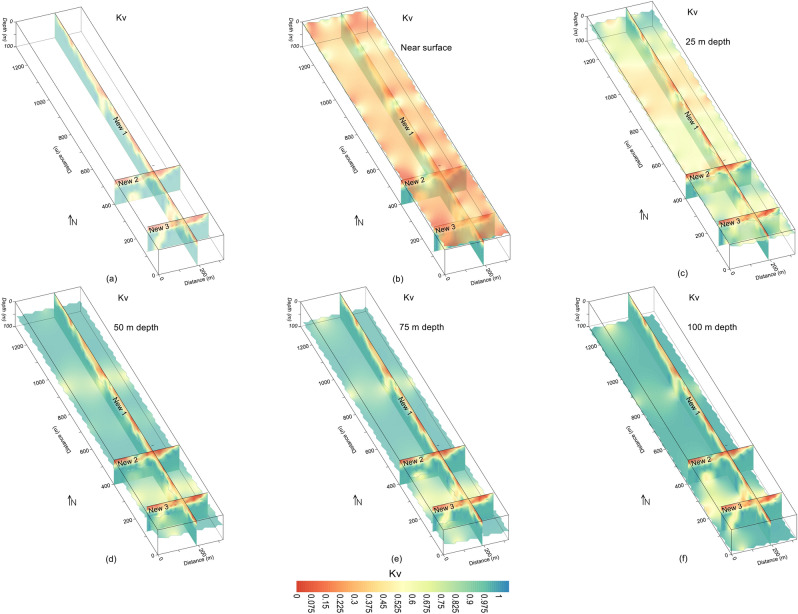
Kv imaging (increasing from red to blue on a color scale) for: (**a**) the integration of three Kv profiles, (**b**) ground surface including (**a**); (**c**) 25 m depth including (**a**); (**d**) 50 m depth including (**a**); (**e**) 75 m depth including (**a**); and (**f**) 100 m depth (bottom) including (**a**).

**Figure 10:**
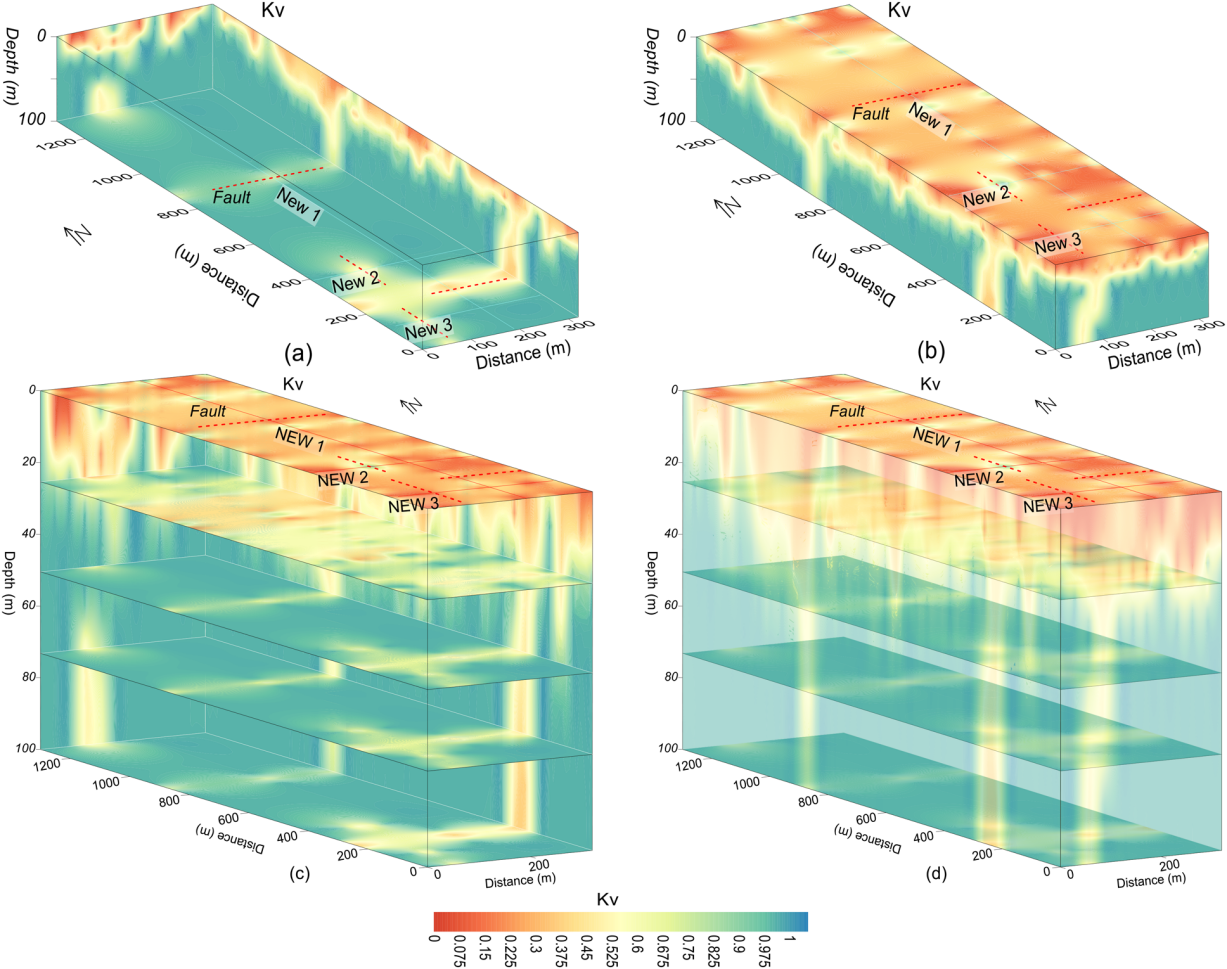
Kv imaging (increasing from red to blue on a color scale) along three profiles (red lines) including faults (dashed red lines) for: (**a**) the inner view of 3D Kv, (**b**) the outer view of 3D Kv, (**c**) different depths from ground surface to 100 m with 3D Kv (inner view), and (**d**) different depths from ground surface to 100 m with 3D Kv (outer view).

**Figure 11:**
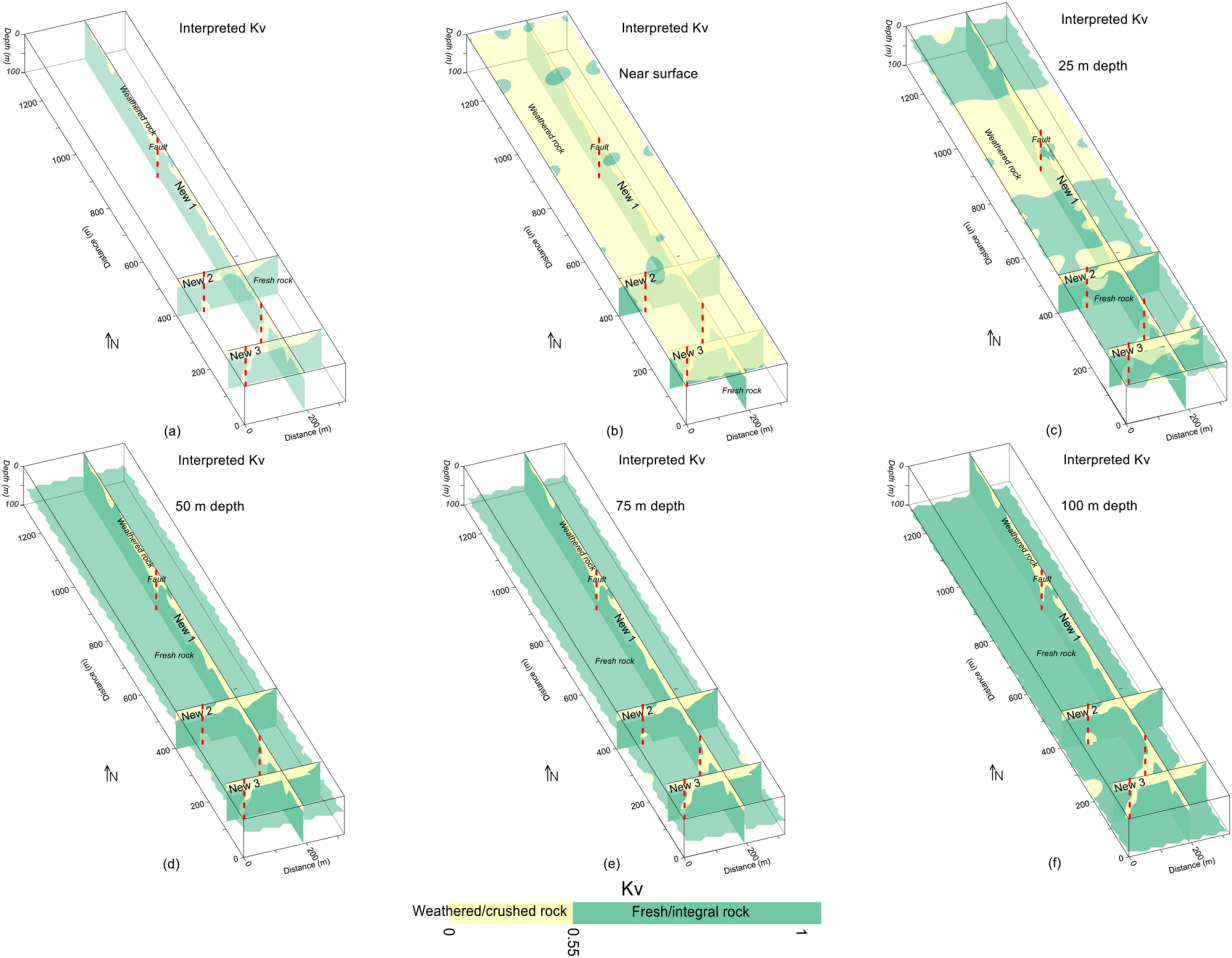
Kv imaging interpreted by crushed/weathered rock (yellow color), integral/fresh rock (green color) and faults (dashed red lines) for: (**a**) the integration of three Kv profiles, (**b**) ground surface including (**a**); (**c**) 25 m depth including (**a**); (**d**) 50 m depth including (**a**); (**e**) 75 m depth including (**a**); and (**f**) 100 m depth (bottom) including (**a**).

**Figure 12:**
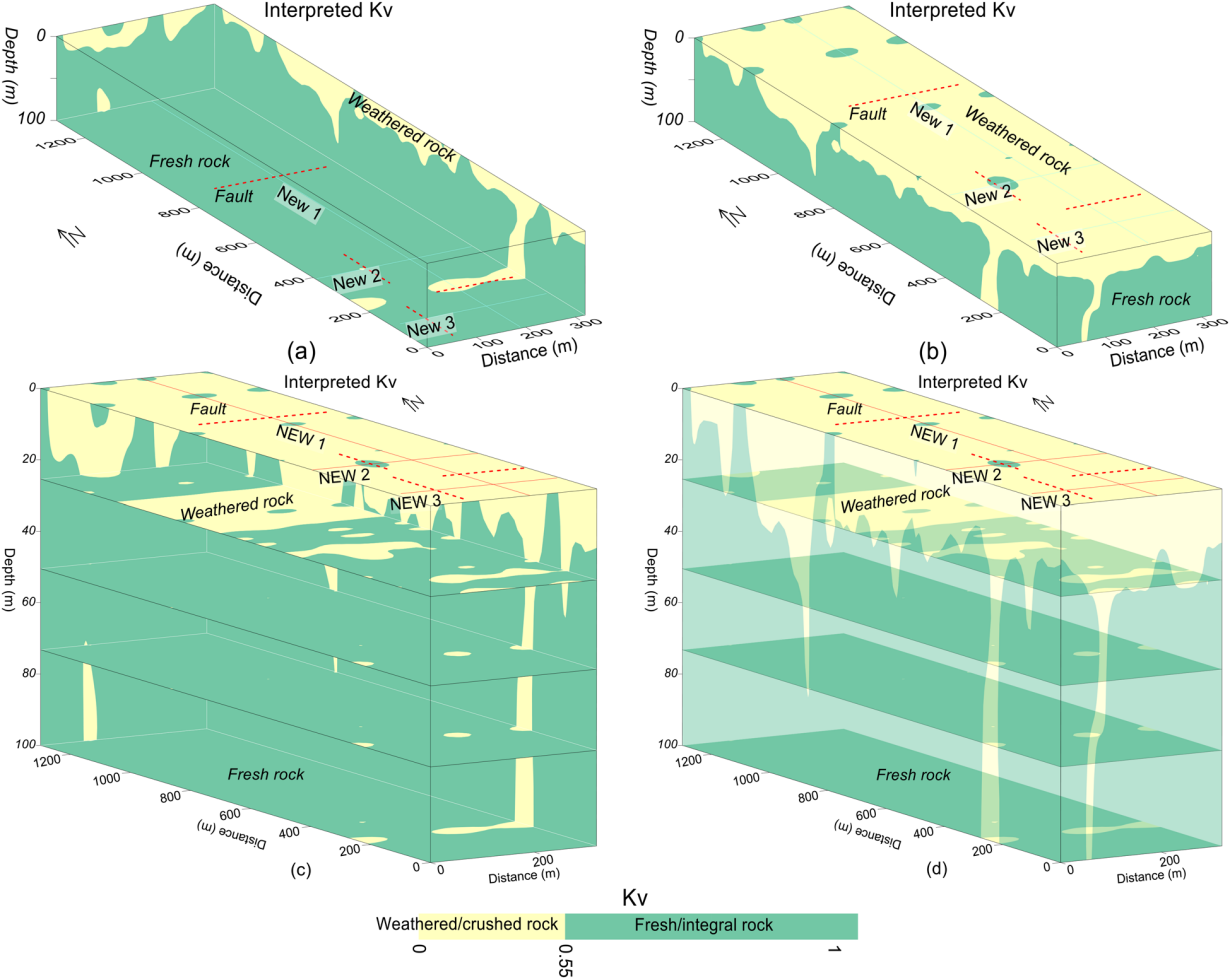
Kv imaging along three profiles (red lines) interpreted by the crushed/weathered rock (yellow color), the integral/fresh rock (green color) and the faults (dashed red lines) for: (**a**) inner view of 3D Kv, (**b**) outer view of 3D Kv, (**c**) different depths including 3D Kv (inner view), (**d**) the outer view of 3D Kv with different depths.

First of all, we used the drilling test data to assess the rock mass quality vertically using Kv at four point locations up to the maximum depth of 40 m only. The evaluation of rock mass quality based on just four drilling tests does not provide accuracy in the interpretation of subsurface geological model, and thus leaves uncertainties prior to the infrastructures development. Afterwards, the quality and strength of subsurface rock mass was evaluated by 2D and 3D mapping of Kv along the same geophysical profiles for investigation depth of 100 m (Figs. 9 and 10). Therefore, a thorough imaging of Kv reduces the uncertainties caused by the limited borehole data and provides more accuracy in the interpretation of subsurface geological model. The subsurface was interpreted by a two layered model based on the specific values range of Kv, such as the weathered/crushed rock (completely, relatively and poorly weathered rock) near the surface with Kv ranging from 0 to 0.55, and the integral/unweathered rock (relatively and completely fresh rock) at the bottom for Kv between 0.55–1.00 (Figs. 11 and 12).

The fresh rock along 2D Kv profile New1 is revealed below 20 m depth especially at 0–200 m, 300–870 m and 900–1300 m distance, whereas two deep zones of poor rock mass quality are also identified along this profile i.e., one such zone of 100 m thickness at distance of 200–300 m, and another zone at distance of 870–900 m with 75 m thickness (Figs. 4d and 11a). Along Kv profile New2, the poor rock mass quality was identified with an average depth of 20 m from the ground surface, this profile detected a deep weathered zone at distance of 90–110 m and depth of 100 m, high quality rock mass was revealed below 20 m depth at distance of 0–90 m and 110–320 m (Figs. 4d and 11a). Similarly, Kv profile New3 evaluated rock mass of high quality below 20 m depth at the distance of 0–40 m and 90–300 m; this profile detected a weathered zone of about 100 m deep at 40–90 m distance, whereas the near-surface weathered layer of poor rock mass quality was delineated with 20 m thickness (Figs. 4d and 11a). The results suggest that, except the identified four deep weathered zones, the rock mass quality is generally good for infrastructures design below 20 m depth. For more detailed view of the subsurface rock mass quality, all 2D Kv models, interpreted for the weathered and fresh rock, were integrated to provide 3D Kv imaging (Fig. 11a). The Kv imaging at different depths (e.g., 0 m, 25 m, 50 m, 75 m and 100 m depth) including the integrated 2D Kv maps is shown in Fig. 11b–f.

Another 3D view of Kv imaging is provided in Fig. 12. The Kv results of Figs. 11 and 12 reveal that the rock mass quality increases with depth. The top surface is almost entirely covered with the weathered/crushed rock of poor quality (Figs. 11b and 12b–d). At 25 m depth, half of the subsurface is still dominant by the weathered/crushed rock especially at 700–1000 m distance along profile New1 and some zones along profiles New2 and New3 (Figs. 11c and 12c, d). However, at 50 m depth and below this depth, the fresh/unweathered rock is dominant which suggests high quality rock mass, and only few small zones of the weathered/crushed rock are identified along profile New3 in the southeast (Fig. 11d–f and 12c, d). Moreover, the Kv results (Figs. 9, 10, 11, 12) are supported by the interpreted 2D/3D ERT maps (Figs. 7 and 8) for rock mass quality evaluation. Hence, a thorough imaging of the subsurface via 2D/3D Kv (Figs. 4b, 9, 10, 11, 12) reduces the uncertainties caused by the limited borehole data and provides more accurate geological model for the development of engineering infrastructures in the study area.

### Faults detection

The fractures/faults provide the weakest foundation for the construction of engineered structures. Hence, detection of such zones is necessary in geotechnical engineering. Groundwater occurrence is mainly associated with the weathered/fractured zones in the hard rock terrains. The fractures/faults can be detected using different geophysical and rock mechanical parameters. Such zones can be detected more accurately using two or more (geophysical/geotechnical) parameters^9^. In this investigation, 2D/3D imaging of ERT and Kv was used for the identification of main faults. The weathered/fractured zones of poor rock mass quality are identified by low values of ERT and Kv. However, the deep weathered/crushed zones were interpreted as the faults/fractures in the project area. We identified several main faults in the study area via 2D and 3D mapping of geophysical (ERT) and geotechnical parameter (Kv) (Figs. 4, 5, 6, 7, 8, 9, 10, 11, 12). Based on a careful observation of all ERT and Kv models, the faults along the deep weathered/crushed zones were delineated by resistivity less than 1000 Ωm (Figs. 4a and 5, 6, 7, 8) and Kv less than 0.55 (Figs. 4b and 9, 10, 11, 12).

Along the deep weathered/crushed rock of the project site, four main faults/fractures were detected with different directions, depths and lengths. One fault of more than 200 m depth was revealed along profiles New1 at 250 m distance, parallel to profiles New2 and New3 in northeast-southwest direction, this fault was detected in southeast part of the study area. The second fault along profile New1 was interpreted at 870 m distance and 75 m depth in northwest and central part of the investigated area parallel to the other two profiles. Next fault of more than 100 m depth is delineated at 100 m distance along Kv profile New2 parallel to profile New1 in northwest-southeast, this fault is identified in southeast of the project site. The last such fault is revealed along profile New3 at 60 m distance parallel to profile New1 in northwest-southeast, this profile is revealed with more than 100 m depth in southeast part of the study area. The results suggest that most of the faults are detected in the southeast part of the investigated area mainly with more than 100 m depth. Therefore, the main faults and the places associated with the fractured zones, delineated by the ERT/Kv models, must be avoided in the development of infrastructures in the project area.

## Discussion

Rock mass quality evaluation is essential for successful construction of the engineering structures in the hard rock terrains. The bearing strength of foundation rocks is mainly determined by the rock mechanical indices. Rock mass integrity coefficient (Kv) and rock quality designation (RQD) are the main geotechnical parameters to evaluate rock mass strength of the subsurface structures prior to the engineering design. Kv is the most efficient rock mass quality index widely used in geotechnical engineering. However, the rock mass quality indices are traditionally determined by the drilling tests. Such tests are expensive, time consuming, cannot be conducted in steep topographic areas, provide low coverage of point measurements only, and thus cannot fulfill the requirements of the planners. On the other hand, geophysical methods are non invasive, economical, user friendly; provide volumetric measurements. Geophysical methods such as electrical resistivity tomography (ERT) have been used in many geotechnical investigations^20–30^. However, the past studies evaluate the subsurface based on geophysical imaging only. In this contribution, we propose a novel approach which can determine the rock mass quality parameters over the entire area by reducing extensive number of borehole tests.

In this work, we make useful empirical correlations between inverted resistivities of the selected ERT data points and Kv values of the limited drilling data. Then, the obtained empirical equations are used to measure Kv for all ERT data points covering the entire area. We acquire two empirical equations for different types of rocks i.e., one equation for the weathered/crushed rock and another for the unweathered/fresh rock. In order to check accuracy of the obtained 2D/3D Kv models, we performed a comparison between the measured and estimated Kv (Table 2) which suggests acceptable accuracy of over 90% matching for most of the data points. The results reveal that ERT and Kv models do not match perfectly with each other, which suggests that Kv provides more accuracy than ERT for rock mass quality evaluation. The bearing strength of subsurface rock improves with an increase in Kv and ERT values mainly from surface to the bottom. The correlation between ERT and Kv reveals that Kv value of 1 (maximum value) remains constant for any value of resistivity over 10,000 Ωm, which suggest that Kv cannot further classify the bearing capacity of fresh rock; however ERT has wide range of resistivity values and can further evaluate the strength of fresh rock. Therefore, ERT can reduce such uncertainty in the interpretation of subsurface geological model caused by narrow range of Kv. The weathered/crushed rock and faults were detected using low values of Kv and ERT. The main faults of the investigated area were interpreted along the deep weathered zones via low values of ERT and Kv. The interpretation of faults via ERT and Kv models reveal that compared with Kv, ERT delineates the faults more accurately, for example, fault detection at 100 m distance along ERT profile New2 is clearer than in Kv model.

**Table 2:** Comparison between the measured and estimated Kv values for the selected data points.

ERT data (selected)				Well data		% Matching
ERT point	Resistivity (Ωm) (selected value)	Estimated Kv′	Depth (m)	Well name	Measured Kv	Kv vs Kv′
New1-41	1008	0.57	25	W1	0.56	98
New1-118	8000	0.95	31	W2	0.97	97
New1-212	505	0.38	10	W3	0.39	97
New2-13	18	0.04	5	W4	0.02	50

**Figure 13:**
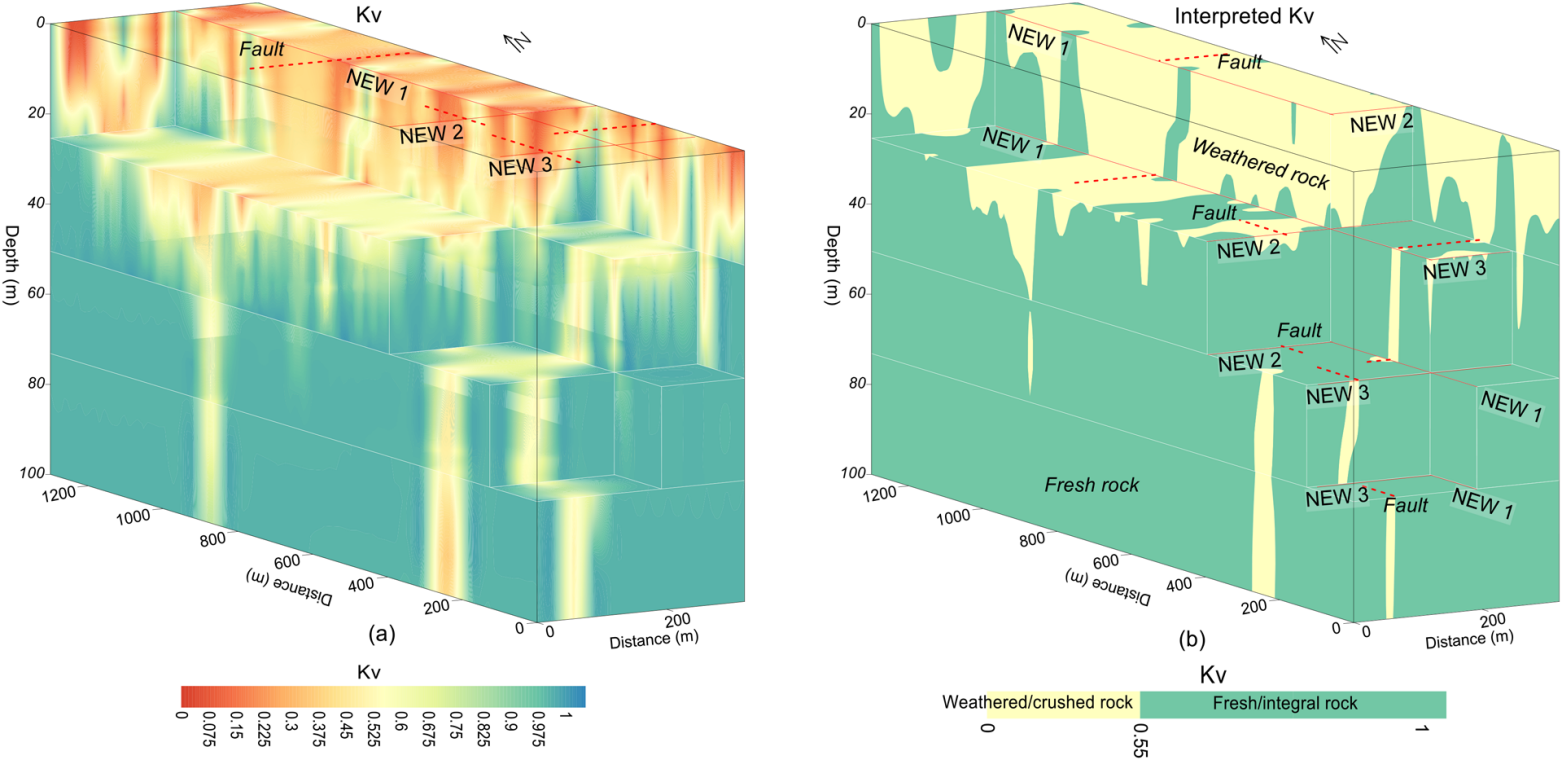
(**a**) Kv imaging (increasing from red to blue on a color scale) along three profiles (red lines) in various dimensions including faults (dotted red lines), and (**b**) Interpretation of a for the weathered/crushed rock (yellow color) and integral/fresh rock (green color).

**Figure 14:**
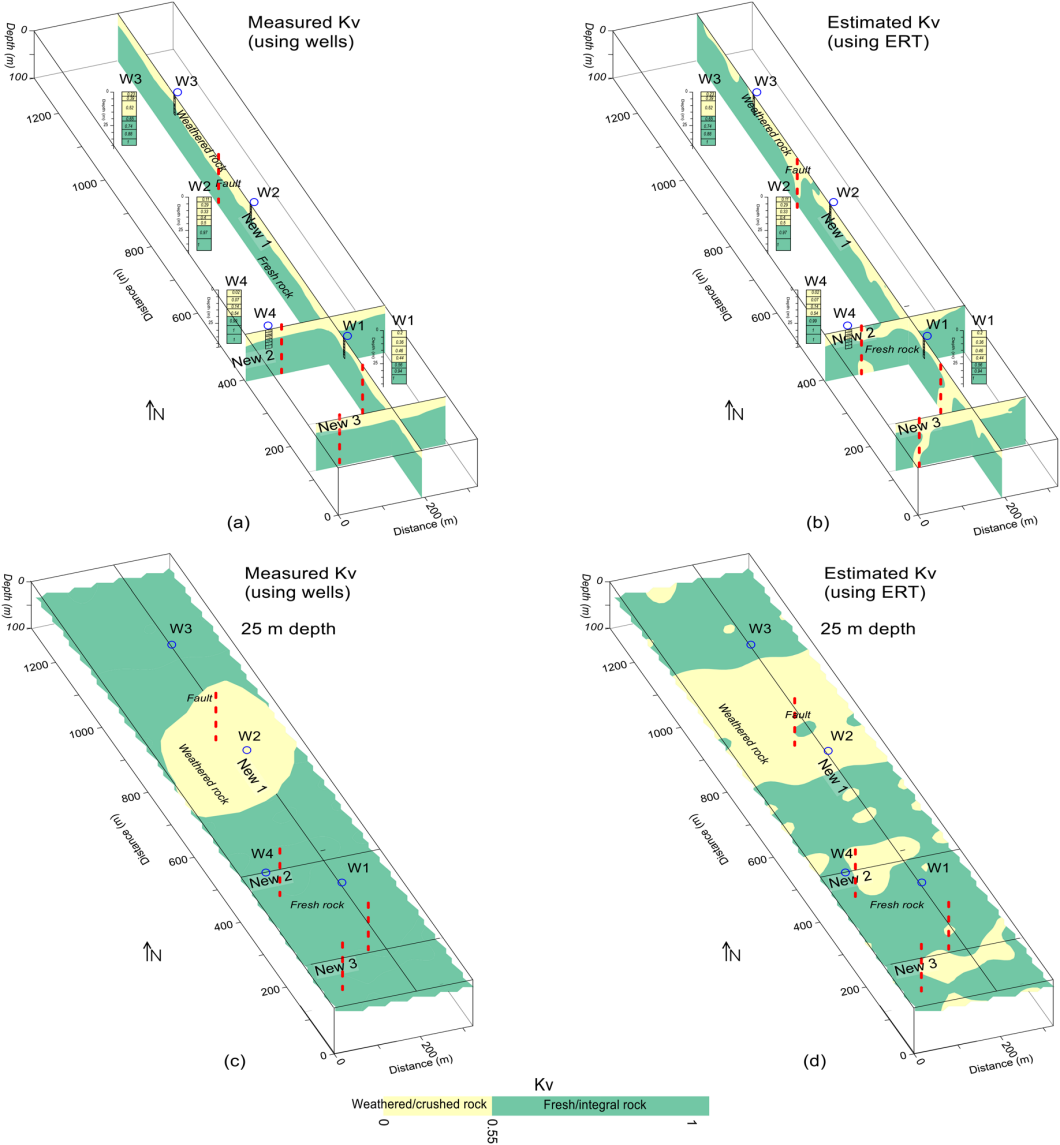
Kv imaging of the subsurface along three profiles interpreted for rock mass quality of weathered/crushed rock (yellow color) and integral/fresh rock (green color) including several faults (dashed red lines), the borehole tests (blue circles and black columns), (**a**) the interpreted 2D Kv models using the drilling test data, (**b**) the interpreted 2D Kv models using the ERT data, (**c**) Kv mapping at 25 m depth using borehole data, and (**d**) Kv mapping at 25 m depth using ERT data.

Compared with the Kv models in Fig. 12, another Kv model in Fig. 13 provides more detailed view of rock mass quality evaluation. Based on our accurate geological Kv model, the construction design was modified for groundwater flow from the faults identified in our model. Further, during the site construction, the groundwater occurrence was found along the same zones as delineated by our faults and the weathered/crushed zones; besides, the groundwater flow from the delineated faults was mainly found towards the water channels of the study area. Figure 14 provides a comprehensive comparison between the Kv results obtained by drilling data and ERT for a two-layered model of weathered and fresh rock. The well data based Kv imaging identifies no fault (deep weathered zone), and provides almost constant mapping of the subsurface layers with the fresh rock at an average depth of 20 m (Fig. 14a). However, the ERT based Kv models, compared with borehole based Kv imaging, reveals several faults and variable mapping of the subsurface distinct layers (Fig. 14b). At 25 m depth, the Kv models obtained by drilling data delineates the rock mass of poor quality along one borehole (W2) and the high quality rock mass along 3 boreholes (W1, W3, W4) (Fig. 14c). However, at the same depth of 25 m, the Kv models generated by ERT (Fig. 14d) provides more detailed mapping of the subsurface geological model, and reveals several weathered zones which are hidden in the drilling based Kv models. Therefore, compared with the inadequate drilling data of 4 boreholes with maximum depth of 40 m, ERT with hundreds of data points for 100 m depth provides far better rock mass quality evaluation. The actual situation of the construction site verified our results obtained by ERT and Kv models. The proposed equations can be used in the areas with similar geological conditions. Using the same methodology, empirical equations can be obtained for areas of any setting.

## Conclusions

In this contribution, we used a non invasive ERT method in geotechnical engineering for rock mass quality evaluation. Conventionally, the drilling approaches are used to obtain geotechnical parameters for the successful construction design of engineering structures. However, such techniques have many limitations and can hardly fulfill requirements of the planners. In order to reduce large number of drilling tests and to obtain thorough insights into the subsurface for rock mass quality evaluation, we introduce our novel approach to provide more accurate geological model of subsurface for infrastructure development. We use this approach to obtain empirical equations via correlations between the selected ERT data and the limited drilling data. Then, the obtained equations are used to obtain Kv imaging along the same ERT models. Thus, we get 2D/3D Kv maps which provide a thorough imaging of the subsurface for rock mass quality evaluation and cover the entire area even where no drilling data are available. The subsurface was evaluated using specific values range of Kv and resistivity, such as the weathered/crushed rock for resistivity less than 1000 Ωm and Kv between 0–0.55, and the unweathered/fresh rock for resistivity greater than 1000 Ωm and Kv ranging from 0.55 to 1.00. The results reveal that rock mass of good quality is found below 25 m depth. The deep weathered/crushed zones were identified as the main faults. The identified faults and the weathered rock were interpreted as the poor rock mass quality, and suggested as unsuitable locations for infrastructures development. The most suitable places of engineering structures were found along the unweathered/fresh rock. It is concluded that the use of geophysical methods, as in this study, can efficiently reduce the ambiguity in the geological model and fill the gaps between the limited data and the accurate geological models.

## Data Availability

Data available on request from the corresponding author.

## Code availability

Software application or custom code support the published claims and comply with field standard.
